# Transforming Medicinal Oil into Advanced Gel: An Update on Advancements

**DOI:** 10.3390/gels10050342

**Published:** 2024-05-17

**Authors:** Rahul Maurya, Lakshminarayana Misro, Thirupataiah Boini, Thulasi Radhakrishnan, Parvathy G. Nair, Sudesh N. Gaidhani, Ankit Jain

**Affiliations:** 1 National Ayurveda Research Institute for Panchakarma, CCRAS, Ministry of AYUSH, Government of India, Cheruthuruthy, Thrissur 679531, India; lakshmi.misro@ccras.nic.in (L.M.); b.thirupataiah@ccras.nic.in (T.B.); thulasi.93@ccras.nic.in (T.R.); parvathynair@ccras.nic.in (P.G.N.); sudesh.g@ccras.nic.in (S.N.G.); 2 Department of Pharmacy, Birla Institute of Technology and Science Pilani, Pilani Campus, Pilani 333031, India

**Keywords:** phytomedicine, herbal gel, polymer, nanogel, hydrogel, stimulus-responsive gel

## Abstract

The present study delves into the evolution of traditional Ayurvedic oil preparations through innovative strategies to develop advanced gel formulations, aiming at amplifying their therapeutic efficacy. Ayurvedic oils have a rich historical context in healing practices, yet their conversion into contemporary gel-based formulations represents a revolutionary approach to augment their medicinal potential. The primary objective of this transformation is to leverage scientific advancements and modern pharmaceutical techniques to enhance the application, absorption, and overall therapeutic impact of these traditional remedies. By encapsulating the essential constituents of Ayurvedic oils within gel matrices, these novel strategies endeavor to improve their stability, bioavailability, and targeted delivery mechanisms. This review highlights the fusion of traditional Ayurvedic wisdom with cutting-edge pharmaceutical technology, paving the way for more effective and accessible utilization of these revered remedies in modern healthcare.

## 1. Introduction

### 1.1. Background

This article describes the intersection of ancient Ayurvedic preparation and modern pharmaceutical advancements. Ayurveda, a centuries-old traditional Indian medicinal system, often utilizes herbal oils for various therapeutic purposes. These oils are known for their healing properties and have been used extensively in traditional treatments. However, in recent times, there has been growing interest in modernizing these traditional Ayurvedic oil preparations to improve their efficacy, convenience of use, and absorption [1]. This interest stems from the desire to combine the ancient wisdom of Ayurveda with contemporary scientific techniques to create more accessible and potent formulations. Gels offer several advantages over oils, such as improved ease of application, reduced greasiness, enhanced skin penetration, and better stability of the active ingredients [2]. This transformation involves understanding the chemical composition of the oils, identifying suitable gelling agents, optimizing formulation parameters, and ensuring the preservation of therapeutic properties while adapting them to the modern pharmaceutical landscape. This article aims to preserve the essence of traditional Ayurvedic medicine while harnessing contemporary scientific knowledge to develop more effective, convenient, and user-friendly formulations with enhanced therapeutic potential [3]. This integration of ancient wisdom and modern innovation promises to unlock new avenues in healthcare, potentially benefiting individuals seeking alternative and holistic approaches to wellness and healing.

### 1.2. Importance of Herbal Formulations

**Figure 1:**
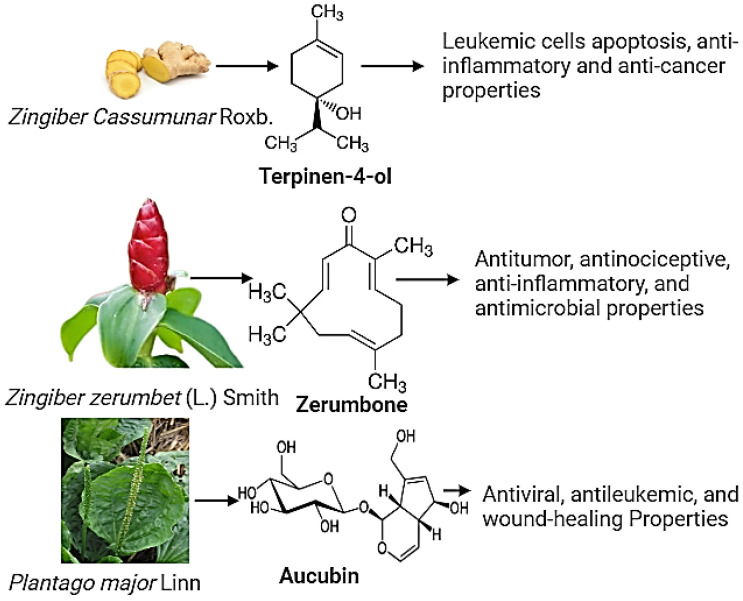
Active phytoconstituents of plants and their clinical applications.

Ayurvedic oil formulations embody profound significance within the holistic healing traditions of Ayurveda, offering a diverse spectrum of therapeutic benefits across various facets of health and wellness (Table 1). These formulations, rooted in ancient wisdom and composed of natural ingredients, showcase their efficacy through many practical applications [4]. For instance, oils consisting of Brahmi (*Bacopa monnieri*) and Ashwagandha (*Withania somnifera*) are renowned for enhancing cognitive functions, promoting mental clarity, and alleviating stress [5]. Similarly, formulations containing ingredients such as Neem (*Azadirachta indica*) and tea tree oil (*Melaleuca alternifolia*) are potent remedies for skin issues, combating acne and eczema, and promoting overall skin health [6,7]. Additionally, oils like mahanarayan oil or ksheerabala oil aid in alleviating muscular discomfort, arthritis, and joint pains, reflecting their efficacy in promoting musculoskeletal health [8]. Moreover, oils containing triphala find applications in supporting digestive health, aiding in detoxification and managing constipation [9]. These examples illustrate Ayurvedic oil formulations’ versatile and practical uses, showcasing their ability to address various health concerns while fostering holistic well-being. Some common phytoconstituents shown in Figure 1, such terpinen-4-ol, show cell apoptosis, anti-inflammatory and anti-cancer properties [10,11,12]. Zerumbone shows antitumor, antinociceptive, anti-inflammatory, and anti-microbial properties [13] The phenolic compound of the plant *Plantago major* Linn shows antiviral, antileukemic, and wound-healing activity [14]. The citrus hystrix DC *Amomum biflorum* Jack shows anti-oxidant activity [15,16]. Zhang et al. reported that certain volatile oils, such as Lavender (*Lavandula angustifolia*) essential oil and *Acorus tatarinowii* essential oil, exhibit notable antidepressant effects while being easily absorbed through the blood–brain barrier. These oils, historically used to alleviate depression and regulate emotions across different cultures, demonstrate promising outcomes in mitigating depressive symptoms with minimal toxicity and side effects. These findings highlight the anti-depressive properties of natural volatile oils and their constituent compounds, elucidating their mechanisms of action in addressing depressive disorders. Additionally, the comparative analysis between these natural volatile oils and conventional antidepressant drugs underscores the potential applications and varied administration routes for integrating these herbal-based therapies into clinical practice. Overall, Zhang et al.’s comprehensive findings offer valuable insights into developing novel and alternative treatments for depressive disorders by harnessing the therapeutic potential of natural volatile oils derived from herbal medicines [17].

### 1.3. Evolution of Gel Formulations

**Figure 2:**
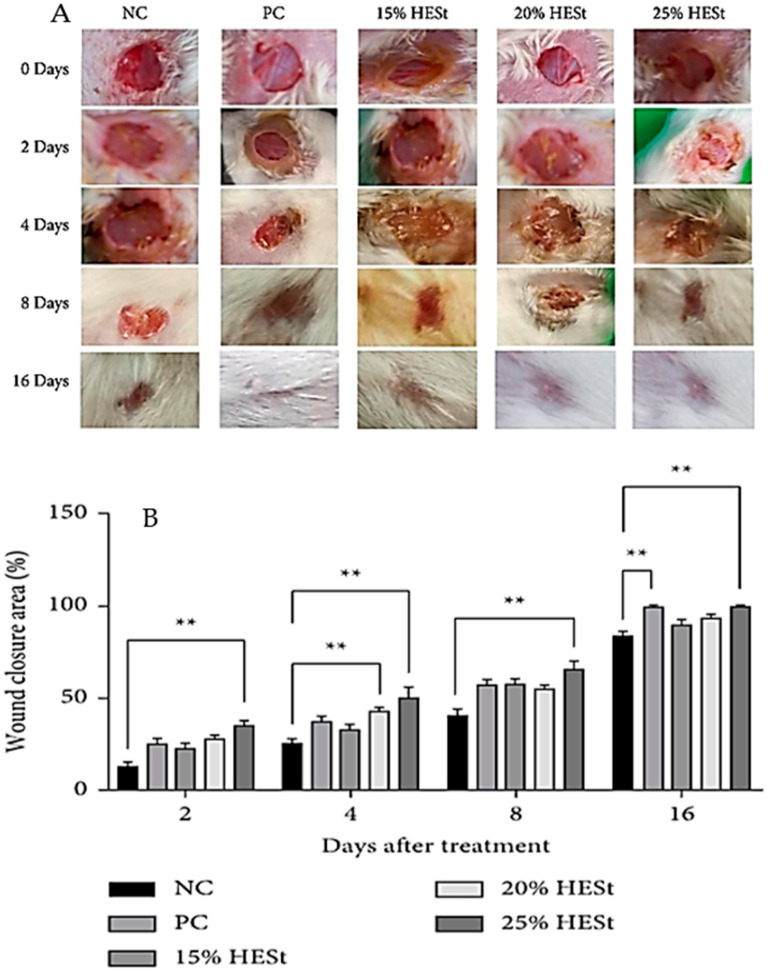
Effect of topical hydrogel of *S. trifasciata* extract (HESt) on the (**A**) % wound closure area. (**B**) Data represent the mean ± SEM ** *p* < 0.05. (NC: negative control; PC: positive control). Image reprinted from Nia Yuniarsih et al. [20] (© 2023) open access article distributed under the Creative Commons Attribution License. (** indicates the comparison between the groups).

The evolution of gel formulations in modern medicine represents a significant advancement in drug delivery systems, offering diverse applications across various medical fields. Over time, these formulations have undergone a remarkable transformation, catering to different therapeutic needs with improved efficacy, stability, and patient compliance. Historically, gels have been used as semi-solid dosage forms in medicine. Traditional hydrogels, composed of water and a polymer network, were primarily utilized for topical applications or as oral formulations. For instance, topical hydrogel formulations containing substances like aloe vera or glycerin have been used for wound healing due to their moisturizing and soothing properties [28]. Abazari et al. discuss the development of polysaccharide-based hydrogels incorporating herbal extracts for wound-healing applications. Figure 2 provides valuable insights into the potential of polysaccharide-based hydrogels integrated with herbal extracts as innovative wound-care solutions, highlighting their structural and functional significance in facilitating the wound-healing cascade [29]. The research conducted by Yuniarsih et al., shown in Figure 2, demonstrates the effects of topical hydrogel of *Sansevieria trifasciata* extract (HESt) on wound healing in mice. The study measured the wound closure area on days 0, 2, 4, 8, and 16 and found that by day 16, the wounds in all the treatment groups were almost completely healed. However, the group treated with HESt at a 25% concentration showed the maximum wound closure effect compared to the other groups. The study reported a direct relationship between the HESt concentration and the % wound closure area. The HESt treatment facilitated significant wound contraction from day 2 to 16 compared to the negative control. Specifically, the 20% and 25% HESt groups exhibited significant wound closure on day 4, while the 15% HESt gel group showed higher closure than the negative control but not significantly. In comparison, the standard drug (octenidine gel) only showed significant wound closure on day 16, with the maximum percentage observed in animals treated with HESt gel at a 25% concentration from day 2 to 16. The HESt 25% group showed a maximum percentage of wound closure at 30.00%, 42.50%, 65.00%, and 99.37% across the observation days, outperforming the standard drug. The study suggests that HESt at a 25% concentration exhibited superior wound-healing activity compared to the standard drug [30].

Modern gel formulations have significantly advanced in their design and composition. Transdermal drug delivery systems, such as gel-based patches, have emerged. Like nicotine or hormone-replacement patches, utilize gels to provide controlled and sustained drug release directly through the skin [31,32]. Wu et al. developed a hydrogel-based soft patch with electrodes designed to facilitate non-invasive iontophoretic drug delivery. The study was conducted on pig skin using dyes as model drugs, successfully demonstrating the feasibility and functionality of the proposed system. The innovation extends the biomedical application and offers a cost-effective solution for non-invasive transdermal drug delivery with closed-loop sensing and treatment capabilities [33]. Ophthalmic gels have evolved to improve ocular drug delivery. These gels offer prolonged contact time with the eye, ensuring better absorption and reduced dosing frequency [34]. For instance, gel-based eye drops for glaucoma or infections provide improved drug bioavailability compared to traditional eye drops. Gel formulations have also seen innovations in controlled drug release systems. Thermosensitive gels have been developed, which change consistency in response to temperature. These gels can be injected as liquids and then form gels at body temperature, providing sustained release of drugs [35]. They find applications in local chemotherapy for cancer treatment. Nanogels, or nanoscale gel particles, have emerged as a frontier in drug delivery. These tiny gel particles loaded with drugs offer precise targeting, enhanced bioavailability, and reduced side effects. They are used in diverse areas such as cancer therapy, delivering drugs directly to tumors while sparing healthy tissues. Modern advancements include hybrid and composite gels, combining different materials to achieve specific properties. For instance, combining natural polymers like chitosan with synthetic materials in wound dressings has resulted in gels with enhanced healing properties and anti-bacterial effects. The evolution of gel formulations in modern medicine has showcased a shift from conventional dosage forms to sophisticated delivery systems, addressing complex medical challenges. These innovations enhance drug delivery, improve patient adherence, minimize side effects, and enable targeted therapy. As technology progresses, the potential for novel gel formulations revolutionizes with traditional medicine to achieve promising medical treatments. Some of the common examples of traditional medicine-based gel formulations are shown in Table 2.

## 2. Traditional Ayurvedic Oil Preparations

### 2.1. Historical Perspective

Traditional medicated oil preparations have a profound historical lineage, deeply embedded in the roots of Ayurvedic practices. Ayurveda, the ancient Indian system of medicine, recognizes the therapeutic potential of herbal oils in promoting health and treating various ailments. The historical texts of Ayurveda, such as the *Charaka Samhita* and *Sushruta Samhita*, extensively document these oils’ formulations, applications, and benefits [56]. Over centuries, these formulations have been refined and passed down through generations, embodying the knowledge acquired from ancient sages and healers. They were utilized in diverse therapeutic practices, including massage therapies (Abhyanga), wound healing, joint ailments, and promoting overall wellness [57].

### 2.2. Key Components and Principles

Ayurvedic oil preparations predominantly use specific base oils known for their therapeutic properties. For instance, sesame oil (*Sesamum indicum*) is used as a base due to its ability to penetrate deeply and pacify vatadosha [58]; however, coconut oil (*Cocos nucifera* Linn) is known for its cooling nature and benefits for Pitta imbalances [59] and ghee (clarified butter) is often utilized for its nurturing and nourishing qualities [60]. Ayurvedic oils are enriched with various herbs and ingredients tailored to address specific health concerns. Some common examples are as follows. Murivenna oil: a classic formulation, combining beneficial herbs like *Pongamia pinnata*, *Aloe vera*, *Piper betel*, *Moringa oleifera*, *Erythrina indica*, *Allium cepa*, *Spermacoce hispida*, *Asparagus racemosus*, *Cocos nucifera*, and *Oryza sativa* traditionally used for treating sprains, fractures, and muscular injuries [61]. Durvadikera: Comprising ingredients such as Durva grass (*Cynodon dactylon*) and medicated oils, it is employed in Ayurvedic therapies for skin diseases like eczema and dermatitis [62]. Bhringamalakadikera oil: blending Bhringraj (*Eclipta alba*) and Amla (*Phyllanthus emblica* Linn) with base oils, it is recognized for its hair-revitalizing properties, used to address hair loss and premature graying [63]. The formulations involve heating base oils with herbal decoctions, infusions, or extracts. This process, called Taila Paka, ensures the infusion of medicinal properties into the oil base. Ayurvedic oils are applied externally through massage techniques or used internally under Ayurvedic guidance. The dosage and application methods vary based on the individual body constitution (dosha) and specific health conditions.

### 2.3. Challenges in Traditional Formulations

Ensuring consistent potency and quality across batches of traditional herbal formulations faces challenges due to variations in ingredients, processing methods, and environmental factors. Integrating traditional knowledge with scientific research and conducting clinical trials is vital to validate the efficacy, safety, and mechanisms of action according to modern healthcare standards. Meeting regulatory compliance and documentation standards for traditional formulations to align with global healthcare regulations poses its own set of difficulties. Moreover, cultural differences, limited awareness, and accessibility to authentic Ayurvedic products hinder their acceptance and usage in regions where traditional medicine is not widely recognized. To overcome these obstacles, a cohesive approach blending traditional wisdom with modern scientific validation is essential. This fusion is crucial in harnessing the therapeutic potential of Ayurvedic oil preparations like murivenna oil, durvadikera, and bhringamalakadikera, facilitating their integration into mainstream healthcare practices worldwide (Table 1). Traditional oil formulations face several challenges, stemming from both technological limitations and evolving consumer demands. One significant challenge is the stability of these formulations, as natural oils can be prone to oxidation, leading to rancidity and a decrease in efficacy over time [64]. Additionally, achieving the desired texture, consistency, and sensory attributes while ensuring compatibility with various skin types poses a challenge. Preservation is another critical concern, as maintaining the shelf-life of oil-based products without compromising their natural profile requires careful balancing of ingredients [65]. Moreover, the demand for sustainable and eco-friendly products prompts challenges in sourcing raw materials ethically and sustainably. Overcoming these hurdles in traditional oil formulations often involves innovative approaches, such as employing anti-oxidant additives, exploring novel extraction methods, and adopting sustainable sourcing practices, to meet the expectations of efficacy, stability, and environmental consciousness in the ever-evolving market [66].

## 3. Advantages of Traditional Medicine

### 3.1. Therapeutic Benefits

A case study was conducted by Varghese et al. on a 61-year-old female who sought treatment due to right knee pain, moderate swelling for five months, limited knee flexion, and difficulty in climbing stairs. Treatment involved a 21-day murivenna bandage application alongside internal medications, including amruthotharam-kashayam, punarnavadikashayam, guggulu-thikthaka-kashyam, yogaraja-guggulu, lakshadi-guggulu, and dasamoolahareetaki-lehyam (Table 1). Following this treatment, the patient experienced pain relief and improved knee joint functionality, highlighting the successful functional restoration achieved within 21 days in this knee osteoarthritis case study [67].

Another case study reported by Gupta et al. for the treatment of vata kantaka, also known as plantar fasciitis, is classified as a nanatmaja-vata-vyadhi, a condition caused by vata dosha. This ailment is typically induced by prolonged exposure to uneven surfaces, leading to heel pain that intensifies during morning walks. Plantar fasciitis affects approximately one in ten individuals during their lifetime, and traditional treatments often fall short of providing satisfactory relief. In a case study, a 35-year-old male patient sought treatment for plantar fasciitis in his left foot. The patient underwent seven sessions of iontophoresis coupled with murivenna-kwatha application on alternate days, alongside daily ten-minute rolling plantar exercises. Remarkably, by the conclusion of the seventh session, the patient experienced significant pain reduction, as evidenced by the visual analog scale dropping from 8 to 1 and the Maryland foot score increasing from 45 to 96. Moreover, during the four-month follow-up, no recurrence of symptoms was reported. Murivenna-kwatha exhibits properties that pacify vata-dosha (functional body units), act as a shulahara (analgesic), and possess shophahara (anti-inflammatory) characteristics. The application of iontophoresis potentially enhances the transdermal absorption of murivenna-kwatha. This combined therapy of iontophoresis with murivenna-kwatha demonstrated notable efficacy in managing and alleviating the pain associated with plantar fasciitis [68].

### 3.2. Need for Innovation

Innovation plays a crucial role in enhancing traditional Ayurvedic oils. Scientific validation through modern research methodologies and clinical trials can validate their efficacy, aiding wider acceptance in healthcare systems. Standardization processes and quality control measures need refinement to ensure consistent potency and quality across different formulations. Modernization through the integration of advanced technologies like nanotechnology or novel delivery systems can improve the bioavailability and efficacy of these oils. Bridging cultural gaps through education and awareness campaigns can expand accessibility and acceptance globally, facilitating the integration of these oils into modern healthcare practices while preserving their traditional therapeutic heritage [69].

## 4. Gel-Based Formulations

### 4.1. Overview of Gel-Based Drug Delivery Systems

**Figure 3:**
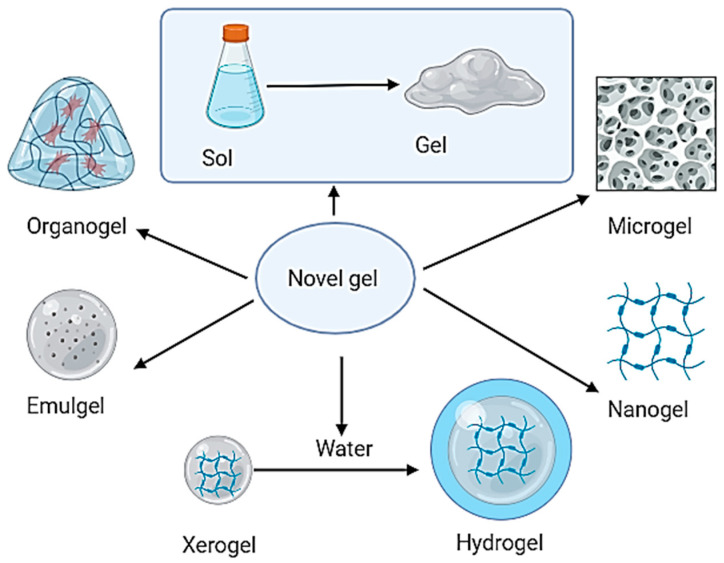
Gel-based drug delivery systems.

Gel-based drug delivery systems represent a versatile and innovative approach to drug delivery, offering a range of applications across various therapeutic areas. These systems utilize gel matrices, typically polymers and solvents, to encapsulate drugs and deliver them to target sites. Hydrogels, organogels, and nanogels are gel formulations that have gained prominence, as shown in Figure 3. Due to their high water content and biocompatibility, hydrogels are extensively used in wound healing, tissue engineering, and as contact lenses due to their ability to maintain a moist environment for optimal healing. Organogels find utility in transdermal drug delivery, enabling efficient skin permeation and controlled release of drugs. Nanogels, with their nanoscale size and tunable properties, offer precise drug delivery, particularly in cancer therapy, allowing for targeted and sustained release of anti-cancer agents to tumor sites. The adaptability and diverse applications of gel-based drug delivery systems make them integral to modern pharmaceutical formulations [70].

### 4.2. Advantages of Gels over Traditional Dosage Forms

Gel formulations offer distinct advantages over traditional dosage forms, contributing to their widespread adoption in modern medicine (Table 2). One key advantage is their versatility in accommodating a wide range of drug types, including hydrophilic and hydrophobic compounds, proteins, and peptides, providing flexibility in drug delivery.

Various synthetic polymers and natural gelling agents (Table 3) have been evolved for the design of gel formulations that provide controlled release kinetics to ensure prolonged drug action compared to conventional dosage forms. Their ability to adhere to mucosal surfaces and skin facilitates targeted and localized drug delivery, minimizing systemic side effects. Furthermore, gels allow for easy administration and application, making them preferable for pediatric and geriatric populations. These advantages underscore the significance of gel-based drug delivery systems in enhancing therapeutic outcomes while ensuring patient convenience [71].

**Figure 4:**
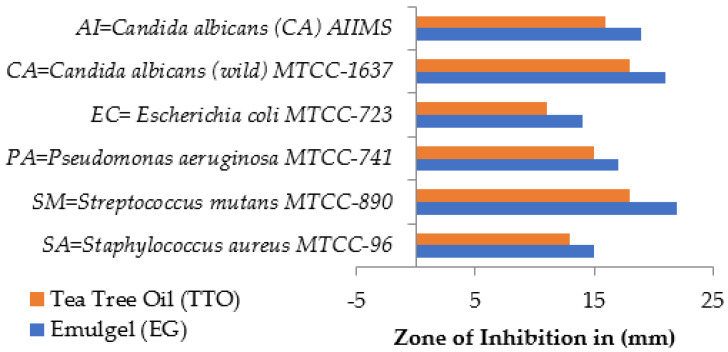
Approximate zone of inhibition of bacterial strains (drawn by findings of Sinha et al. [95]).

Sinha et al. prepared a nanoemulsion (NE) of tea tree oil (TTO) using high-energy emulsification techniques. The NE was then formulated into an emulgel (EG) using pH-sensitive polymer carbopol 940. The EG demonstrated superior inhibitory effects against selected microbial strains compared to pure TTO, as shown in Figure 4. The EG showed a favorable pH matching human skin, efficient skin permeation, and appropriate viscosity and texture parameters for topical application. Furthermore, the EG proved to be non-irritating and exhibited consistent properties over 90 days [95].

### 4.3. Applications of Gels in Pharmaceuticals

The success stories of gel-based drug delivery systems span diverse pharmaceutical applications. For instance, gels containing therapeutic agents have revolutionized applications in pain management, bacterial diseases, fungal diseases, anti-oxidants, antiviral, etc. (Table 2) [96]. Additionally, innovative nanostructured lipid gels and hydrogels are advancing targeted drug delivery in cancer therapies, demonstrating promising outcomes in delivering chemotherapeutic agents directly to tumor sites while minimizing systemic toxicity [97]. These success stories highlight the efficacy of gel-based drug delivery systems and their diverse applications across multiple therapeutic domains, establishing their pivotal role in modern pharmaceutical formulations [98].

Tadic et al. studied the effects of creams and emulgels containing immortelle extract and hemp oil on healthy human skin. Various parameters were measured to evaluate the products’ impact, including the stratum corneum hydration (measured via electrical capacitance), skin barrier function (evaluated through TEWL—transepidermal water loss), tolerability (assessed by changes in EI—erythema and irritation), and skin pH. The research included a group of volunteers within a narrow age range. Both immortelle extract and hemp oil have shown positive effects on the skin. The research demonstrated that a night cream with immortelle extract improved the skin hydration by 64.4% and reduced the TEWL by 10% after one hour of application. Hemp seed (*Cannabis sativa* L.) oil also displayed promising results by significantly reducing the TEWL and improving subjective feelings of dryness and itchiness in volunteers with atopic dermatitis over eight weeks. The TEWL, a measure indicating skin permeability and integrity, plays a crucial role in assessing skin health. Lower TEWL values correspond to healthier skin, while increased values suggest skin damage. The finding suggests that the application of all the tested creams and emulgels led to decreased TEWL values, indicating an enhancement of the skin barrier. The findings suggest that the tested preparations, containing immortelle extract and hemp oil, effectively improved the skin hydration, reduced the TEWL, and potentially enhanced the skin barrier function [99].

## 5. Integration of Ayurvedic Principles into Gel Formulations

Integrating Ayurvedic principles into gel formulations involves harmonizing ancient wisdom with modern pharmaceutical technology. Ayurveda emphasizes the balance between the doshas (vata, pitta, kapha) and the importance of individual constitution (prakriti) in maintaining health. When adapting Ayurvedic principles to gel transformations, considerations are made to select compatible base materials and techniques aligning with these principles (Table 3) [100]. For instance, the choice of base oils in gels like sesame or coconut, known for their dosha-balancing properties. The processing techniques employed, such as Taila Paka (medicated oil preparation), aim to retain the medicinal properties of herbs while creating stable gel formulations [101]. Preserving the therapeutic properties of Ayurvedic ingredients during the transformation into gel formulations remains a focal point [102]. Techniques involving microencapsulation or nanoemulsions are explored to maintain the stability and bioavailability of these constituents in the gel, preserving their therapeutic efficacy [103]. The goal is to ensure that the gel formulations derived from Ayurvedic principles retain medicinal benefits while enhancing their delivery and application [104]. A crucial aspect of integrating Ayurvedic principles into gel formulations involves preserving the holistic healing approaches inherent in Ayurveda. Examples shown in Table 2 illustrate the promising therapeutic potential of plant bioactive transformation into gel form. The holistic approach considers the overall impact on an individual’s well-being, ensuring that the gel formulations derived from Ayurvedic principles align with the holistic healing philosophy, offering comprehensive wellness benefits beyond mere symptomatic relief [105].

## 6. Emerging Techniques for Gel Formation

### 6.1. Nanotechnology in Gel Development

**Figure 5:**
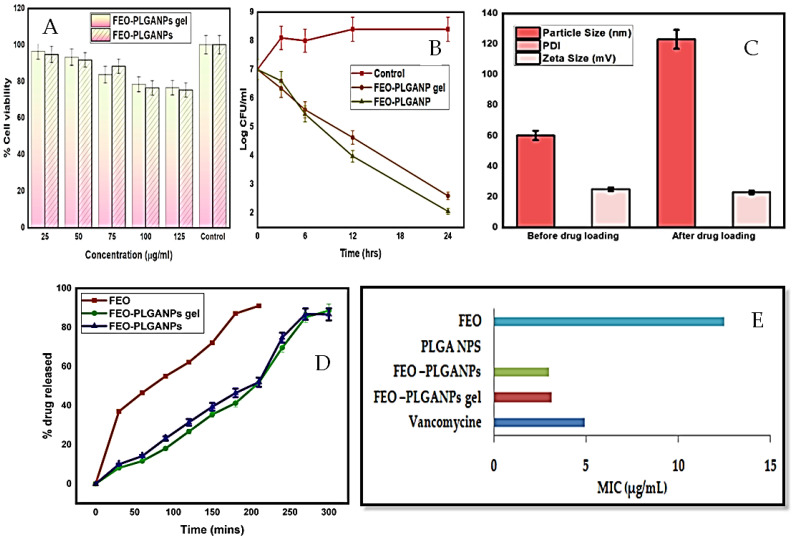
Comparative study of FEO–PLGANPs gel and FEO–PLGANPs (**A**) cell viability assay. (**B**) Time–kill assay. (**C**) Particle size, polydispersity index and zeta potential of FEO–PLGANPs. (**D**) In vitro % drug release of FEO, FEO–PLGANPs, and FEO–PLGANPs in pH 6.8. (**E**) Minimum inhibitory concentration (MIC) of fennel essential oil-loaded PLGA nanoparticles (FEO-PLGANPs) gel, FEO-PLGANPs, PLGANPs, and fennel essential oil (FEO) against *S. aureus* (MTCC 10787) bacteria (results are adopted from Alam et al.) [108]. Image reprint from Alam et al. [108]. (© 2022) open access article distributed under the Creative Commons Attribution (CC BY) License.

Nanotechnology has emerged as a groundbreaking approach to gel formation, revolutionizing drug delivery systems. Nanogels, with their nanoscale size and unique properties, offer precise control over drug release and enhanced therapeutic efficacy. These nanogels utilize advanced nanotechnology techniques like self-assembly or crosslinking to encapsulate drugs, providing controlled release kinetics. Their small size allows efficient penetration into tissues, offering promising solutions in various medical fields, such as oncology, dermatology, and ophthalmology, by facilitating targeted and sustained drug delivery [106,107]. Alam et al. reported a comparison between the anti-bacterial activities of pure fennel (*Foeniculum vulgare*) essential oil (FEO), FEO-loaded PLGA nanoparticles (FEO-PLGANPs), and FEO-PLGANPs gel against *S. aureus* (MTCC 10787), as shown in Figure 5. The combination of PLGANPs and FEO exhibited a synergistic effect, enhancing the anti-microbial activity significantly. The FEO-PLGANPs gel displayed notably higher anti-bacterial potency compared to the pure FEO, with statistically significant differences (*p* < 0.05) in their effectiveness against *S. aureus*. This enhanced activity was attributed to various factors, like the occlusive properties, specific drug-carrier interactions, and close contact facilitated by the small size of nanoparticles. Both the FEO-PLGANPs and FEO-PLGANPs gel exhibited similar minimum inhibitory concentrations (MICs) against *S. aureus*, showcasing their efficacy with MIC values of 3.00 and 3.12 µg/mL, respectively, while the naked FEO had an MIC of 12.5 µg/mL. Overall, the developed FEO-PLGANPs and FEO-PLGANPs gel demonstrated effectiveness against *S. aureus*, were deemed environmentally safe, and indicated potential applicability in skincare products. The finding suggests that the combination of FEO and PLGANPs displayed enhanced anti-bacterial properties, with the FEO-PLGANPs gel exhibiting superior efficacy compared to pure FEO [108]. Other findings also show the anti-bacterial effects of essential oils. The MIC values of origanum oil (*Origanum vulgare, thymol chemotype*) against methicillin-resistant *S. aureus* and *S. epidermidis* strains, as well as strains of *S. aureus* and *S. epidermidis*, varied from 0.063% to 0.125% (*v*/*v*). Similarly, tea tree (*Melaleuca alternifolia*) oil, basil oil (*Ocimum basilicum* Linn.), origanum oil (*Origanum vulgare* L.), and thyme oil (*Thymus vulgaris*) showed MIC values against *S. aureus* at concentrations of 0.5%, 2%, 0.12%, and 0.25% (*v*/*v*) [10], respectively [109].

### 6.2. Encapsulation Strategies

Encapsulation strategies play a pivotal role in gel formation, ensuring the protection of active compounds and controlled release. Microencapsulation and nanoencapsulation involve enveloping drug molecules within micro- or nano-sized particles, enhancing their stability and bioavailability. For instance, in pharmaceuticals, microencapsulation of drugs within gel matrices provides sustained release profiles, improving drug efficacy and patient compliance. These strategies safeguard sensitive compounds, preserving their integrity during storage and delivery, thereby expanding the scope of drug formulations, including those derived from traditional medicinal practices [110,111].

Mendez et al. reported nanoencapsulation strategies, including conformal coating and layer-by-layer coating, particularly for the immuno-protection of islets of Langerhans. Conformal coating, a non-spherical encapsulation method, reduces diffusion distance and implant volume. However, concerns persist regarding potential damage to encapsulated cells during the multi-step process and uncertainties about its clinical application [112]. While some studies suggest a lower immune-protective capacity compared to hydrogel microcapsules, recent strategies have shown the potential of conformal-coating technology [113,114,115]. Layer-by-layer coatings involve alternating layers of polymers with opposite charges on the cell surface, improving biomaterial/cell ratio and therapeutic molecule diffusion. Promising results have been observed in rodent studies, and a recent non-human primate study, where layer-by-layer encapsulated pancreatic islets exhibited 100% survival for 150 days after xenotransplantation [116,117,118]. Despite the advantages, polymers used in nanoencapsulation may be less biocompatible than hydrogels. Adding a second type of coating to attenuate the shapes and complement the system could be beneficial in nanoencapsulation. Conversely, some studies propose that larger microspheres (1.5 mm) have fewer immune system cells and fibrotic processes, suggesting a potential advantage of larger sizes. In conclusion, the size of microencapsulation systems has diverse implications for cell viability and immune responses, and ongoing research aims to strike a balance between optimizing the size for efficient nutrient exchange and minimizing the adverse effects on the encapsulated cells.

**Figure 6:**
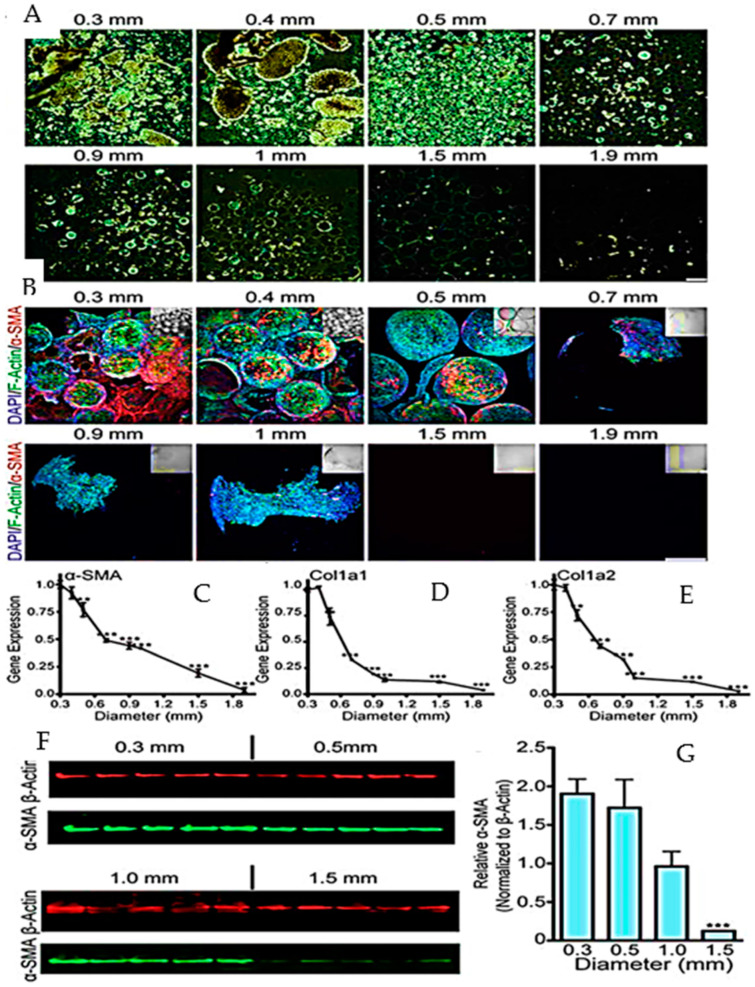
Study of (0.5 mL SLG20) alginate spheres (0.3–1.9 mm) implanted in the intraperitoneal space of C57BL/6 mice for 14 days and evaluated for fibrosis upon retrieval. (**A**) Dark phase contrast pictures of recovered spheres show a noticeable reduction in cellular proliferation as the sphere size increases. (**B**) Staining cell nuclei, F-actin, and α-SMA in myofibroblast cells. (**C**) Study of fibrotic markers (α-SMA) using q-PCR based expression: (**D**) collagen (1a1) and (**E**) collagen 1a2 (size 0.3–1.9 mm) spheres normalized to relative expression (300 μm sized spheres). (**F**) The expression of α-SMA in cellular overgrowth on microspheres was assessed by semi-quantitative Western blotting. Each mouse was assigned a band number ranging from 1 to 5. (**G**) ±SEM, *n* = 5. All study n = 3, Bonferroni multiple comparison correction with one-way ANOVA—** *p* < 0.001, *** *p* < 0.0001. Image reprinted from Mendez et al. [112]. and it is an open-access article (Copyright © 2021 by authors) distributed under the terms and conditions of the Creative Commons CC BY license permission Reference number 240219-015475.

The experiment conducted by Veiseh et al. shown in Figure 6 reported that the study investigates the impact of the alginate sphere size on cellular deposition and fibrosis formation. SLG20 alginate spheres, each with a volume of 0.5 mL, were produced in various sizes (ranging from 0.3 to 1.9 mm) and implanted into the intraperitoneal space of C57BL/6 mice. These spheres were retained for 14 days, after which they were retrieved and analyzed for fibrosis levels. The results reveal a noteworthy trend: an increase in sphere size corresponds to a substantial reduction in cellular overgrowth. The dark phase contrast images of the retrieved spheres exhibit a significant decrease in cellular overgrowth as the size of the spheres increases. Z-stacked confocal images stained for cellular nuclei (DAPI), F-actin (phalloidin), and myofibroblast cells (α-Smooth Muscle Actin, α-SMA) further support this observation. Quantitative PCR (q-PCR) analysis of the fibrotic markers, including α-SMA, collagen 1a1, and collagen 1a2, directly on spheres of different sizes corroborates the trend. The expression levels of these markers decrease with increasing sphere size, particularly when compared to the 300 μm sized spheres. Semi-quantitative Western blot analysis of α-SMA expression in cell overgrowth on microspheres provides additional evidence. The plotted band intensities from the Western blot images further demonstrate a reduction in α-SMA expression with larger sphere sizes. The study concludes that increasing the alginate sphere size leads to a substantial decrease in cellular deposition and fibrosis formation, with implications for the design and application of such spheres in various biomedical contexts. The presented data offer valuable insights into optimizing the alginate sphere size to minimize adverse cellular responses in practical applications [119].

### 6.3. Novel Polymers and Excipients

Advancements in gel formation involve the utilization of novel polymers and excipients, expanding the range of potential formulations. Some of the natural gelling agents are shown in Table 3. These innovative materials offer improved characteristics such as better gel strength, controlled release, and enhanced bioavailability. For example, incorporating biocompatible polymers like pectin, agar, alginate, and chitosan into gel matrices enhances their stability and biodegradability, which is ideal for sustained drug release in tissue engineering or wound-healing applications. Excipients like cyclodextrins or lipid-based carriers enable solubility enhancement of poorly soluble drugs, broadening the scope of drug delivery in pharmaceutical formulations [120,121,122].

### 6.4. Role of Surfactants in Gel Formation

Surfactants play a crucial role in gel formation by modulating formulations’ surface tension and viscosity. They aid in stabilizing emulsions, enhancing drug solubility, and controlling gel consistency. For instance, surfactants facilitate the dispersion of hydrophobic drugs in topical gel formulations in the aqueous phase, improving their bioavailability upon application. Surfactants also influence gel rheology, impacting factors such as spreadability and adhesion. Tailoring surfactant selection and concentrations allows fine-tuning of the gel properties, optimizing their performance in various pharmaceutical and biomedical applications [123,124].

## 7. Enhancing Efficacy through Formulation Modifications

### 7.1. Bioavailability Enhancement

Formulation modifications of gels aim to enhance bioavailability, ensuring maximum drug absorption and efficacy. For instance, incorporating penetration enhancers or absorption promoters in transdermal gel formulations facilitates drug permeation through the skin barrier, improving bioavailability. Additionally, the use of nanogels with their high surface-area-to-volume ratio enhances drug solubility and absorption, thereby increasing bioavailability. Bioavailability is significantly improved by optimizing the gel composition and employing innovative techniques, such as lipid-based formulations or nanoemulsions, ensuring better therapeutic outcomes for patients [125,126].

### 7.2. Controlled Release Mechanisms

**Figure 7:**
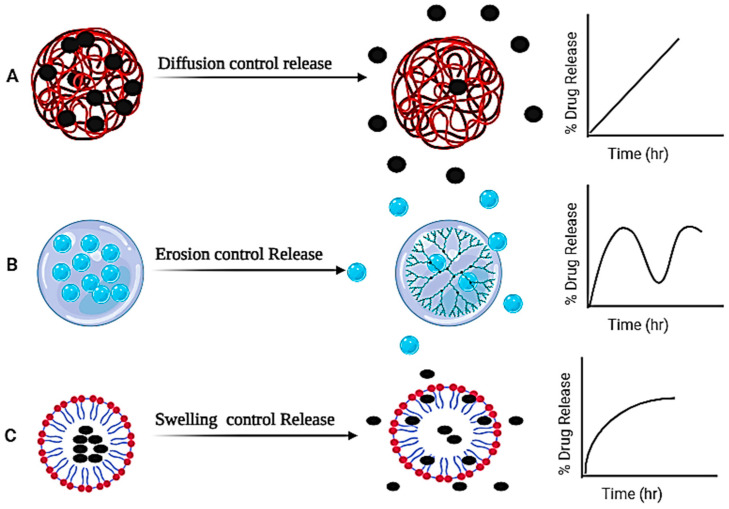
Different release mechanisms: (**A**) diffusion-controlled (**B**) erosion-controlled and (**C**) swelling-controlled.

Gel modifications offer controlled release mechanisms, ensuring sustained and targeted delivery of drugs, as shown in Figure 7. For example, hydrogels containing drug-loaded nanoparticles enable controlled release, maintaining therapeutic drug levels over an extended period. Similarly, stimuli-responsive gels respond to external cues like temperature, pH, or enzymatic activity, triggering controlled drug release at specific sites within the body. By tailoring gel formulations to control the rate and duration of drug release, therapeutic efficacy is optimized, reducing the dosing frequency and minimizing side effects [127,128]. The release of oil from hydrogels involves intricate mechanisms that are crucial for designing effective systems in applications of controlled drug delivery. One primary mechanism is diffusion, where oil molecules permeate the polymeric matrix of the hydrogel through concentration gradients. Zhao et al. illustrated that the hydrogel containing *Perilla frutescens* L. essential oil (PLEO) exhibited prolonged release characteristics. The release profile, modeled using the Peppas–Sahlin approach, revealed that Fickian diffusion predominantly governed the release mechanism [129]. Responsive hydrogels, exhibiting swelling–deswelling behavior in response to stimuli, represent another key mechanism. For instance, a pH-responsive hydrogel may encapsulate oil in a swollen state, and upon exposure to a specific pH range, it releases the oil due to changes in the polymer structure. Deng et al. explore the behavior of hydrogel particles in response to pH fluctuations. At a pH range from 3 to 5, the hydrogel particle demonstrates uniform dimensions within the range of 0.53–0.73 μm. However, with an increase in pH from 5 to 7, a notable expansion in the hydrogel particle size is noted, ranging between 5.77 and 18.33 μm. This size modulation underscores the sensitivity of hydrogel particles to changes in pH and emphasizes facilitating the controlled release of lavender (*Lavandula angustifolia*) essential oil [130]. Hydrophobic interactions between the polymeric chains and oil molecules also play a role, as hydrophobic regions attract and retain oil within the hydrogel structure. Kwan et al. documented the substantial hydrophobic interaction between flavor oil and the polymeric network of pectin and whey protein isolate, emphasizing its pivotal role in controlling the release behavior of the hydrogel. The results underscore the importance of pH at 4.0 in establishing a polymer network conducive to hydrophobic interactions, leading to the effective entrapment and release of flavor oil [131]. External stimuli swelling and erosion of the polymer also regulate the release property of the hydrogel. Biodegradation, through enzymatic activity, can lead to the breakdown of the hydrogel matrix, facilitating oil release over time. Saidi et al. discussed the swelling properties of hydrogel were influenced by variations in the molecular weights of Polyethylene glycol (PEG)- and polyethylene glycol–polycaprolactone (PCL). Hydrogels follow non-Fickian diffusion, but as the PCL concentration increased and PEG molecular weights decreased, the release mechanism shifted entirely from non-Fickian to Fickian diffusion. This shift was attributed to the increased cross-link density and PCL concentration leading to decreased swelling degree and network flexibility [132]. Ali et al. developed a pH-sensitive hydrogel by crosslinking *Salvia spinosa* seeds with citric acid. The hydrogel demonstrated its highest swelling capacity in deionized water compared to buffers of pH 1.2, 6.8, and 7.4. Additionally, the hydrogel sustained drug release following zero-order kinetics and a non-Fickian diffusion mechanism [133]. Additionally, swelling and environmental triggers, such as temperature or pH changes, can be harnessed to modulate drug release from hydrogels [134,135]. Setapa et al. introduced novel two-phase drug release model that incorporates device swelling, and its effectiveness is evaluated using experimental data from three distinct BC-g-P(AA) hydrogels. The established model proves capable of accurately estimating drug release in swelling drug delivery devices. Numerical results indicate that initial diffusion coefficients for all the hydrogels are lower than the later diffusion coefficients, primarily attributed to changes in the device pore size during swelling [136]. The in vitro drug release profiles of swelling-controlled and erosion-controlled systems are inherently nonlinear due to the intricate and dynamic processes governing drug release from these matrices. In swelling-controlled systems, the nonlinear behavior arises from the dynamic changes in the matrix’s swelling characteristics, altering the effective drug release area, diffusion path length, and drug-related domain over time [137]. Similarly, in erosion-controlled systems, the nonlinearity stems from the progressive degradation of the matrix, leading to variations in the structural integrity and surface area available for drug release. The interplay between polymer swelling, erosion dynamics, and environmental factors contributes to the complexity of these systems, resulting in nonlinear drug release kinetics [138]. In contrast, diffusion-controlled systems exhibit linearity in their drug release profiles due to the consistent and predictable nature of drug diffusion through the matrix. In diffusion-controlled systems, drug release is primarily governed by Fickian diffusion, leading to a linear relationship between time and cumulative drug release [139]. The distinct mechanisms in swelling-controlled and erosion-controlled systems introduce variability and nonlinearity into the drug-releasee behavior. These mechanisms collectively provide a versatile toolkit for tailoring hydrogel properties to specific applications, allowing for controlled and efficient drug release [140], as depicted in Figure 7.

### 7.3. Addressing Stability Issues

Modifications to gel formulations address stability challenges encountered in drug delivery systems. Strategies such as encapsulating sensitive compounds within gel matrices or using stabilizers prevent drug degradation and maintain formulation integrity. For instance, liposomal gel formulations protect bioactive compounds from degradation by environmental factors, preserving their stability and efficacy. These modifications ensure the prolonged shelf life and sustained effectiveness of the gel-based drug delivery systems [141,142]. Formulations play a crucial role in safeguarding oils from degradation by providing a protective matrix that minimizes exposure to external factors. One common method involves the encapsulation of oils within polymeric micro- or nanostructures, forming a barrier against oxygen, light, and other destabilizing elements [143]. For instance, the use of biodegradable polymers such as poly (lactic-co-glycolic acid) (PLGA) or starch-based polymers for encapsulation not only shields the oils such as clove oil but also facilitates controlled release [144]. Additionally, polymeric coatings on oil droplets can prevent oxidation by acting as a physical barrier [145]. Polymers like alginate or chitosan are employed for their film-forming properties, creating a protective layer around omega-3 rich oils to minimize the oxidation [146]. Leong et al. investigated the oxidative stability and anti-oxidant properties of microencapsulated kenaf (*Hibiscus cannabinus* L.) seed oil (MKSO) produced through co-extrusion technology during accelerated storage. The study utilized a combination of sodium alginate, methoxyl pectin, and chitosan as shell materials. The results demonstrated that the MKSO exhibited significantly lower oxidation levels compared to bulk kenaf seed oil (BKSO). The research concluded that the system effectively protected kenaf seed oil from lipid oxidation, delaying the degradation of natural anti-oxidants during storage [147]. Overall, formulations provide versatile solutions for enhancing the stability and shelf life of oil-loaded systems through their protective and controlled release properties.

### 7.4. Customization for Specific Therapeutic Targets

Gel formulation modifications allow customization of specific therapeutic targets, catering to diverse medical needs. Tailoring gel compositions with specific drug combinations or adjusting the release kinetics meets the requirements of various therapeutic applications. For instance, in ocular drug delivery, mucoadhesive gels are customized to adhere to the eye surface, providing sustained drug release and improving treatment outcomes for eye-related conditions [148]. Similarly, personalized formulations for targeted drug delivery to tumors in cancer therapy demonstrate the potential of gel modifications in addressing specific therapeutic targets, thereby maximizing treatment efficacy [149].

**Figure 8:**
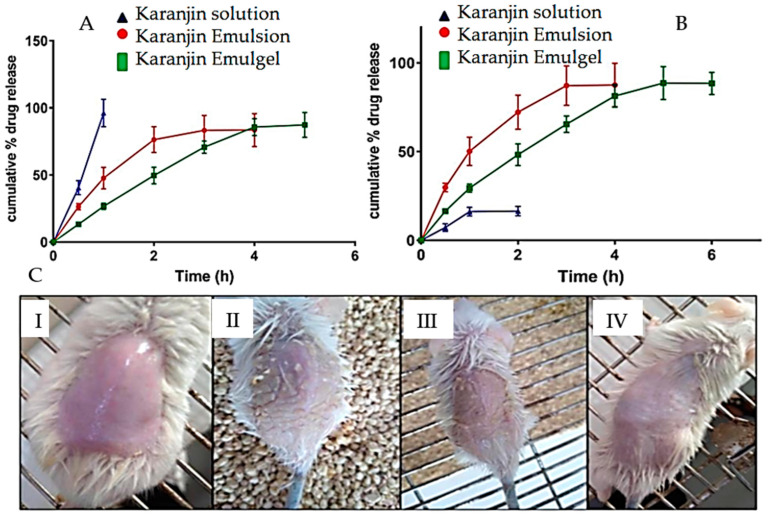
Study of Karanjin emulgel: (**A**) in vitro release from Karanjin from solution, emulsion and emulgel; (**B**) ex vivo permeation of Karanjin from solution, emulsion and emulgel; (**C**) animal skin morphology; (**I**) normal control; (**II**) disease control; (**III**) Karanjin solution and (**IV**) Karanjin emulgel. Image reprint from Desai et al. [150]. and is an open-access article copyright Author(s) 2023 distributed under Creative Commons CC-BY 4.0.

Desai et al. developed Karanjin emulgel for the treatment of psoriasis. Karanjin is the major bioactive compound present in *Pongamia pinnata*. The emulsion containing Karanjin was prepared using peppermint oil and incorporated into a gel base carbopol 940. The emulsion (KE5) had a droplet size of 110.4 ± 1.56 nm, with the zeta potential of −40.9 ± 1.11 mV, entrapment efficiency of 92.12 ± 2.8%, and a creaming volume of 98.1 ± 0.4. Short-term stability studies indicated the stability of KE5 for 30 days. The pH and viscosity of the emulgel were 7.31 and 8060 cp, respectively. In vitro release studies revealed a slow release of Karanjin, with 95.36% released in 6 h. Ex vivo permeation studies reported 88% permeation of Karanjin in 5 h from the emulgel compared to 16.52% permeation from control. In vivo studies showed a significant improvement in the psoriasis area and severity index (PASI) score upon the application of the Karanjin emulgel. These results underscore the potential efficacy of the emulgel in enhancing the effectiveness of Karanjin against psoriasis (Figure 8) [150].

## 8. Case Studies on Successful Transformations

### 8.1. Specific Examples of Ayurvedic Oil-to-Gel Conversion

Comparative studies between traditional Ayurvedic oil preparations and their novel gel counterparts provide valuable insights into their efficacy and patient outcomes. For instance, the study reported by Salem et al. focused on evaluating the skincare potential of selected *Apiaceous* essential oils traditionally used in cosmetics. Coriander (*Coriandrum sativum*) essential oil shows the highest inhibition of elastase, tyrosinase, collagenase, and hyaluronidase compared to other oils like *Foeniculum vulgare*, *Pimpinella anisum*, and *Cuminum cyminum* L. Gas chromatography-mass spectrometry (GC-MS) analysis revealed coriander oil’s predominant oxygenated monoterpenes, particularly linalool (81.29%). Notably, formulations using coriander oil cream and coriander essential oil-loaded lipid nanoparticles (CEOLNs) demonstrated a remarkable ability to mitigate in vivo UV-induced skin photoaging, reducing the markers of oxidative stress (MDA), inflammation (COX-2, PGE-2), matrix degradation (MMP-1), and signaling pathways (JNK, AP-1). These formulations also significantly boosted the skin collagen content and increased the expression levels of proteins involved in skin repair (TGFβ, TGFβII, SMAD3). This study concludes that CEOLNs exhibit anti-wrinkle properties, showcasing promising potential in counteracting external signs of aging [151].

Shukr and Metwally formulated a carbopol 940 gel with oils of lemon grass (*Cymbopogon citratus*) and thyme (*Thymus vulgaris*) and tested how well it worked against methicillin-resistant *Staphylococcus aureus* (MRSA) skin diseases. The results showed that this gel had anti-microbial activity and did not irritate the skin, which suggests that it is safe to use on the skin [152].

### 8.2. Clinical Outcomes and Patient Responses

A 30-year-old female patient sought Ayurvedic treatment at IPGT&RA Hospital for persistent heel pain in her right foot, stemming from a previous injury to the Achilles tendon due to a road traffic accident. Despite initial wound treatment that resulted in healing within two months, the patient continued to experience intermittent heel pain, impacting her daily life and occasionally requiring analgesics. Examination revealed mild swelling, stiffness, and tenderness at the Achilles tendon insertion point, with no evidence of bony lesions on foot X-rays. The healed scar showed no discoloration or significant deformity, but mild swelling persisted on the heel’s posterior aspect. Palpation elicited tenderness and soft swelling, and painful dorsiflexion was noted, although other foot movements were within normal limits. The diagnosis of Achilles tendinopathy was established, and treatment involved a unique approach using Murivenna oil Parisheka for one month. The application of Murivenna oil led to a notable reduction in pain and stiffness within 15 days and complete relief within a month. The range of foot movements also improved significantly during this period. This case study showcases the promising role of Murivenna oil in managing Achilles tendinopathy and soft tissue injuries due to its enhanced skin permeability and potent anti-inflammatory effects [22].

### 8.3. Clinical Trials, Safety and Efficacy

The safety and efficacy of a herbal product called *aayudh advance*, which contains essential oils, were evaluated in a randomized control trial with 60 patients (30 individuals in each group) who had COVID-19. Following 14 days of therapy, every single patient in the *aayudh advance* group had fully recovered, demonstrating a recovery rate that was 15.38% greater than that of the Standard of Care group treated alone. Furthermore, the viral load was significantly reduced (*p* < 0.05), as shown by a notable rise in the CT values of the E-gene and RDRP genes. There were no reports of side effects, and the supplementary therapy did not interfere with any drugs. With a better rate of recovery in the treatment arm compared to the control arm in mild symptomatic COVID-19 patients, the study shows that *aayudh advance* is both safe and more effective in lowering the viral load [153]. Mali et al. compared Arimedadi (herbal) oil (Table 1) to chlorhexidine gluconate (0.2% *w*/*v*) mouthwash in terms of the anti-plaque efficacy. Group A was the control group, Group B was the chlorhexidine gluconate group, and Group C was the Arimedadi oil group. A total of 45 patients with mild to moderate gingivitis were randomly allocated to each of these three groups. The participants used their assigned mouthwash twice a day for 21 days after the baseline examinations and tooth scaling. The plaque index (PI) and gingival index (GI) were assessed on days 7, 14, and 21. Up until day 21, there were noticeable improvements in gingival health relative to the control group in both the chlorhexidine and Arimedadi groups. The gingival health of the Arimedadi group was significantly better than that of the chlorhexidine group beginning on day 14. The study suggested that Arimedadi oil is a safe and effective alternative to chlorhexidine gluconate as an adjuvant to mechanical plaque management [154].

## 9. Commercial Potential of Patented Plant-Derived Medicinal Oil-Based Formulation

The commercial prospects of patents associated with essential oil-based gels and other herbal gels are highly promising due to their innovative formulations and unique properties. These patents signify groundbreaking advancements in the fusion of essential oils and herbal extracts, presenting distinctive solutions that hold significant commercial appeal (Table 3) [155]. As consumers increasingly prioritize natural and holistic products, the patented gels, boasting organic ingredients and therapeutic benefits, align seamlessly with this growing demand [156]. The potential applications of these patented formulations span a wide spectrum, from skincare to pain relief, tapping into diverse market segments. The inherent uniqueness of these patented gels not only differentiates them in a competitive market but also positions them as cutting-edge, high-quality products. Furthermore, the global focus on health and wellness amplifies the marketability of these gels, presenting opportunities for brands to establish a strong presence both domestically and internationally [157]. Patents, serving as a testament to the products’ ingenuity, not only protect intellectual property but also provide a solid foundation for effective marketing strategies, ensuring their visibility and credibility in the commercial landscape [158].

Oliveira et al. conducted a study on nanotechnological inventions involving essential oils, revealing that the primary application areas are the food industry (57%), pharmaceutical industry (22%), agro-industry (13%), and the chemical industry (8%)]. A similar investigation by Silva et al. reported that 31% of oil nanoencapsulation applications are in the food industry, while 26% are in the pharmaceutical industry for drugs and herbal products [159]. Overall, the commercial utility of patents related to essential oil-based and herbal gels lies in their ability to meet the evolving needs of consumers, capitalize on emerging market trends, and establish a distinctive presence in the competitive marketplace [160].

## 10. Challenges and Future Perspectives

**Figure 9:**
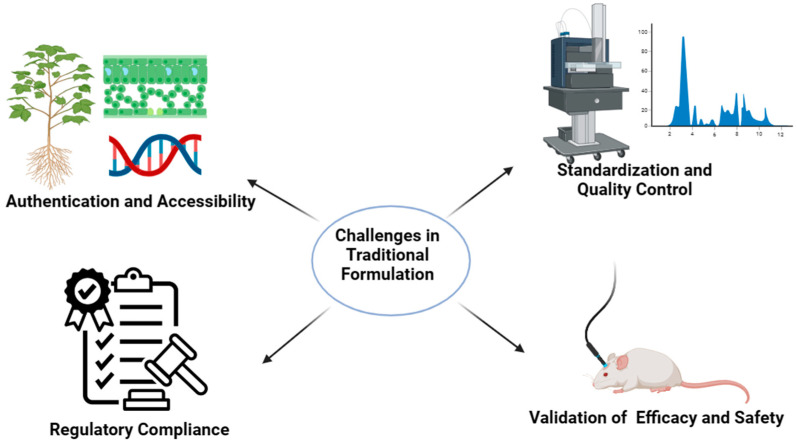
Challenges and future perspectives.

One of the significant challenges in integrating Ayurvedic principles into gel formulations involves navigating regulatory hurdles. Harmonizing traditional Ayurvedic practices with modern regulatory frameworks poses complexities due to differences in documentation, validation standards, and acceptance criteria across various regions. For instance, achieving the regulatory compliance of Ayurvedic-based gel formulations involves stringent documentation, safety assessments, and efficacy validations, often requiring extensive clinical trials and data submissions, as shown in Figure 8. Ensuring alignment with established pharmaceutical regulations while preserving the essence of traditional knowledge creates a challenging landscape for manufacturers and researchers seeking to introduce these formulations into mainstream healthcare systems [161,162]. Standardization and quality control present critical challenges in developing Ayurvedic-based gel formulations. Maintaining consistent potency, purity, and quality across batches is essential but complex due to variations in raw materials, processing methods, and environmental factors. For instance, standardizing the composition and dosage of herbal extracts or oils within gel matrices while ensuring batch-to-batch consistency remains a significant challenge. Implementing robust quality-control measures and analytical techniques to authenticate and quantify the active compounds in these formulations becomes imperative to meet quality standards and ensure patient safety [163]. Ethical considerations surrounding traditional knowledge preservation, fair practices, and cultural respect emerge as pivotal factors in developing and commercializing Ayurvedic-based gel formulations. Ethical challenges encompass issues related to intellectual property rights, equitable sharing of benefits with local communities, and respecting Indigenous knowledge systems. For example, ethically sourced raw materials and ensuring fair compensation to traditional practitioners or local communities for their knowledge pose ethical dilemmas. Striking a balance between commercial interests, ethical practices, and cultural sensitivities remains a challenging yet essential aspect of developing and utilizing Ayurvedic-based gel formulations [164]. Despite the challenges, the future of Ayurvedic-based gel formulations holds promise, offering opportunities for innovative research and advancements (Figure 9). Exploring novel technologies like nanogels or smart delivery systems to enhance targeted drug delivery and bioavailability presents exciting avenues. Further research into advanced analytical techniques and pharmacokinetic studies to validate these formulations’ efficacy, safety, and mechanisms of action is crucial. Collaborations between traditional medicine practitioners, pharmaceutical experts, and regulatory bodies can foster a better understanding of Ayurvedic principles and facilitate their integration into evidence-based modern healthcare. Future directions also include establishing global standards for quality control, harmonizing regulatory frameworks, promoting ethical practices, and fostering a conducive environment for developing and utilizing Ayurvedic-based gel formulations [165,166,167].

## 11. Conclusions

This summary focuses on innovative approaches aimed at upgrading traditional Ayurvedic oil preparations into advanced gel formulations to augment their therapeutic efficacy. Ayurvedic oils have long been utilized for their healing properties, but their transformation into gel-based formulations represents a modernization strategy to enhance their therapeutic potential. This evolution involves leveraging scientific advancements to modify these traditional oils into more advanced and accessible gel formulations. The primary objective is to improve their application, absorption, and effectiveness in addressing various health conditions. By encapsulating the beneficial components of Ayurvedic oils within gel formulations, these novel strategies aim to enhance their stability, bioavailability, and targeted delivery, thereby maximizing their therapeutic impact. This research seeks to bridge the gap between traditional Ayurvedic knowledge and modern pharmaceutical advancements, promising a paradigm shift in the utilization and effectiveness of these time-honored remedies in healthcare (Table 4).

## Figures and Tables

**Table 1:** Different traditional formulations, their compositions and applications.

Ayurvedic Oil	Composition	Application	Ref.
Arimedadi oil	*Rubia cordifolia*, *Acacia catechu*, *Sesamum inidicum*, *Syzygium aromaticum*	Anti-microbial	[18]
Mahanarayan oil	*Clerodendrum phlomidis* Linn F, *Eclipta alba* Hassk, *Aegle marmelos* Corr, *Cedrus deodara (Roxb)* Loud, *Solanum xanthocarpum* Schrad and Wendle, *Saussurea lappa* C B Clarke, *Rubia cordifolia* Linn, *Teramnus labialis* Spreng, *Phaseolus trilobus* Ait, *Cyperus rotundus* Linn, *Mesua ferrea* Linn, *Erythrina indica* Lam, *Stereospermum suaveolens* DC, *Trianthema portulacastrum* Linn, *Inula racemosa* Hook f, *Pluchea lanceolata* Oliver and Hierm, *Parmelia perlata* (Huds) Ach, *Desmodium gangeticum* DC, *Oroxylum indicum* Vent, *Glycyrrhiza glabra* Linn	Anti-inflammatory	[19]
Ksheerabala oil	*Go-Ksheera* (cow milk), *Sida cordifolia* and *Tila Taila*	Neurological, heart disease, anti-inflammatory, and hepato-protective activity	[20]
Triphala	*Terminalia bellirica*, *Terminalia chebula* and *Emblica officinalis*	Immunomodulator	[21]
Murivenna oil	*Pongamia pinnata*, *Aloe vera*, *Piper betel*, *Moringa oleifera*, *Erythrina indica*, *Allium cepa*, *Spermacoce hispida*, *Asparagus racemosus*, *Cocos nucifera*, and *Oryza sativa*	Anti-inflammatory	[22]
Bhringamalakadikera oil	*Eclipta alba*, *Emblica officinalis*, *Glycyrrhiza glabra*, Cow milk	Hair loss, baldness, graying of hairs	
Amruthotharam kashayam	*Zingiber officinalis*, *Tinospora cordifolia*, *Terminalia chebula*	Anti-inflammatory, metabolic disorders	[23]
Punarnavadi kashayam	*Boerhavia diffusa*, *Zingiber officinale*, *Cidrus deodara*, *Commiphora mukul*	Hypothyroidism	[24]
Gugguluthikthaka kashyam	*Adhatoda vasica*, *Azadirachta indica*, *Acorus calamus*, *Aconitum heterophyllum*, *Alpinia calcarata*, *Anetham graveolens*, *Commiphora mukul*, *Celastrus paniculatus*, *Cedrus deodara*, *Cuminum cyminum*, *Cyprus rotundus*, *Curcuma longa*, *Cyclea peltata Embelia ribes*, *Holarrhena antidysenterica*, *Piper longum Piper nigrum*, *Piper brachystachyum*, *Picrorhiza kurroa*, *Plumbago zeylanica*, *Solanum indicum*, *Rubia cordifolia*, *Saussura lappa*, *Scindapsus officinale*, *Tinospora cordifolia*, *Trichosanthes dioica Trachyspermum roxburghianum*, *Zingiber officinale*, *Semecarpus anacardium*	Anti-inflammatory	[25]
Yogaraja guggulu	*Piper longum*, *Trachyspermum ammi*, *Carum carvi*, *Embelia ribes*, *Apium leptophyllum*, *Cuminum cyminum*, *Cedrus deodara*, *Piper chaba*, *Elettaria cardamomum*, *Saindhava lavana*, *Saussurea lappa*, *Pluchea lanceolata*, *Tribulus terrestris*, *Coriandrum sativum*, *Terminalia chebula*, *Terminalia bellirica*, *Emblica officinalis*, *Cyperus rotundus*, *Zingiber officinale*, *Piper nigrum*, *Piper longum*, *Cinnamomum zeylancium*, *Vetiveria zizanoides*, *Hordeum vulgare*, *Taxus wallichii*, *Cinnamomum tamala*, *Commiphora wightii*	Neurological, musculoskeletal disorders	[26]
Lakshadi guggulu	*Cissus quadrangularis*, *Withania somnifera*, *Azadirachta indica*, *Sida alba*, and *Terminalia arjuna. C. quadrangularis*	Anti-fungal, anti-bacterial, anti-oxidant, analgesic and anti-inflammatory	[27]

**Table 2:** Some examples of plant-based bioactive materials used in the preparation of gel formulations.

Carrier	Material Used in Gel Formulation	Method of Preparation	Therapeutic Agent	Characterization	Application	Ref.
Nanoethosome gel	Ethosome- Lipid (1–3%) Ethanol (20–40%) Propylene glycol (20%) Extract (2%) Gel- Carbopol 940 Triethanolamines Nanoethosomes	Ethosome- Cold method followed by sonication Gel- Stirring method	*Achillea millefolium* L. (AM) hydroalcoholic extract	Ethosome- EE: 90 ± 0.74% Size: 240 nm PDI: 0.24 ± 0.017 Zeta: −31.1 mV Gel- pH: 5.5–6.1 Dp: 79.8% Viscosity: 4520–4760 cP	Anti-aging	[36]
Microemulsion gel	Microemulsion- Tween 80 Isopropyl alcohol Clove oil Water Gel- Carbopol 934 Microemulsion Glycerene	Microemulsion- Phase titration method Gel- Stirring method	Clove (*Syzygium aromaticum*) oil	Microemulsion- Size: 14.41 nm PDI: 0.0113 Zeta: 0.73 mV Gel- pH: 6.27 DR: 98.5 ± 0.35% Viscosity: 12.87 m·pas/s	Anti-fungal	[37]
Gel	Gel-Carbopol 940, Sodium carboxymethylcellulose	Gel- Stirring method	*Mentha longifolia* essential oil	Pseudo-plastic shear-thinning behavior Inhibitory activity against: *Candida albicans* (ATCC90028 and MTCC277)	Candidiasis	[38]
Cream	Oil phase- Liquid paraffin (5.0 mL) Stearic acid (4.8 g) Coconut oil (3.0 mL) The aqueous phase- Triethanolamine (1.5 mL) Glycerin (5.0 mL) Citric acid (0.4 g) Methylparaben (0.1 g) Water Aloe vera gel (2–8 mL)	Mixing	Aloe vera, tomato powder	pH: 7.3–7.6 Spreadability: 9–13 Acid values: 5.6–7.2	Skin nourishment	[39]
Oleogel	Aerosil, paraffin, and olive oil	Stirring method	Thyme (*Thymus vulgaris*) essential oil	DL: 0.1% *w*/*w* MIC: *Candida albicans* (ATCC 60193) 0.01 to 0.3%	Anti-fungal	[40]
Niosome gel	Neosome- Non-ionic surfactants and cholesterol Gel- Mucilage of *Lallemantia royaleana* Benth and carbopol	Niosome– Ether injection method Gel- Stirring method	Ibuprofen, *Lallemantia royaleana Benth*	Neosome- Size: 3.2 ± 0.75 µm EE: 46 ± 1.0% DR: 45.5 ± 1.2 % Yield: 75.0 ± 0.87 Zeta: −66.32 mV Gel- Q: 1.548 ± 1.09 mg cm^−2^ Jss: 8.256 ± 1.15 mg cm^−2^ h^−1^ Log Kp: 0.433 ± 0.76 Edema inhibition: 18.66%	Anti-inflammatory	[41]
Neosome gel	Niosome- Span and cholesterol Gel- Carbopol 940	Niosome- Reverse-phase evaporation technique Gel- Stirring method	Rosmarinic acid	Niosome- EE: 65% ± 3.99. DR: 70% in 12 h Gel- pH: 6.4 ± 0.159 Content uniformity: 90% Spreadability: 19.6 ± 1.118 g cm/s DR: 49.81 ± 1.76% MIC: 31.25 µg/mL *S. aureus* (MTCC 96), 31.25 µg/mL *Propionobacterium acne* (MTCC 1951)	Anti-bacterial	[42]
Hydrogel	Chitosan	Ionic gelation method	Eugenol oil	Graft yield (*wt %*): 21.7 ± 1.0% pH: 3–9	Anti-oxidant	[43]
Hydrogel	Calcium alginate, and CaCl_2_	Emulsion fabrication methods	Cumin (*Cuminum cyminum* L.) seeds essential oil	DR: 96.02 ± 0.96% (SGF) and 10.65 ± 1.23% (SIF)	Anti-fungal, anti-bacterial and anti-oxidant	[44]
Starch hydrogels	Carbopol and starch	Mixing	Patchouli (*Pogostemon cablin* Benth.) essential oil	Viscosity: 15.016 ± 59 cP Spreadability: 4.02 ± 0.34 g·cm/s pH: 6.81 to 7.23.	Anti-microbial, anti-inflammatory and anti-cancer	[45]
Nanoemulsion hydrogel	Alginate and CaCl_2_	Mixing	Cinnamon (*Cinnamomum verum*) essential oil	Size: 146.20 ± 39.28 nm Zeta: −33.8 ± 0.72 mV	Anti-microbial	[46]
Hydrogel membranes	Esterification of polyvinyl alcohol (PVA) with starch and glutaraldehyde	Esterification	Essential oils of clove oil (*Syzygium aromaticum*), Oregano oil (*Origanum vulgare*), and tea tree (*Melaleuca alternifolia*) oil	ZOI: 39 ± 0.57 mm (*MRSA*) and 37 ± 0.29 mm (*E. coli*). Ts: 19.36 MPa WVTR: 36.22 g/m^2^h MRC: 95.50%	Anti-bacterial	[47]
Liposomal gel	Liposome- Soya lecithin and cholesterol (1:1) Gel- Hydroxyethylcellulose	Liposome- Freeze–thaw method stirring Gel- Stirring method	*Eucalyptus camaldulensis* essential oil	EE: 95 ± 0.57% MIV: 0.125 mL Size: 157.66 ± 0.57 nm	Anti-fungal	[48]
Liposome	Liposome- Soya phosphatidylcholine and cholesterol Gel- Hydroxyethylcellulose	Liposome- Film method and sonication Gel- Stirring method	*Santolina insularis* essential oil	EE: 80.00 ± 0.55 Size: 63 ± 12 nm	Anti-viral	[49]
Nanoliposome gel	Liposome- Soya lecithin, phytosterol, and α-tocopherol Gel- Xanthan gum	Liposome- Sonication, homogenization Gel- Stirring method	Eucalyptus (*Eucalyptus globulus*), tea tree (*Melaleuca alternifolia*) oil, clove (*Syzygium aromaticum*) oil, and coconut (*Cocos nucifera* L.) oil	Liposome- Size: 50–115 nm Zeta: −34 to −43 mV PDI: 0.190 ± 0.027 EE: 95% Gel- pH: 5.5–6.0 MIC: 15 (*E. coli*), 15.5 *(B. subtilis*)	Anti-microbial	[50]
Liposomal gel	Liposome- Soya lecithin, phytosterol, and α-tocopherol Gel- Carbopol		Tretinoin (TRE) and zedoary turmeric oil (ZTO)	Liposome- Size: 257.41 ± 7.58 nm Zeta: −38.77 ± 0.81 mV PDI: 0.10 ± 0.04 EE: % 64.63 ± 1.00 (ZTO), 90.33 ± 0.72 (TRE) DL: % 9.09 ± 0.14 (ZTO), 1.43 ± 0.02 (TRE) Gel- Skin permeation: 11.9533 ± 1.3934 μg/cm^2^ (ZTO) and 6.7033 ± 1.3803 μg/cm^2^ (TRE) reduction in vaginal epithelial mitotic activity	Psoriasis	[51]
Nano-transferosome in situ gel	Transferosome- Soya lecithin and Tween 80 Gel- Deacetylated gellan gum	Transferosome- Thin-layer evaporation technique Gel- Stirring method	Voriconazole-Clove (*Syzygium aromaticum*) oil	Transferosome- Size: 102.96 nm Zeta: −38.77 ± 0.81 mV PDI: 0.10 ± 0.04 EE: % 71.70 DL: % 9.09 ± 0.14, 1.43 ± 0.02 Gel- ZOI: 21.76 mm DR: 82.5% Dp: 5.4-fold increase	Anti-fungal	[52]
Transferosomal gel	Transferosome- Soya lecithin and cholesterol	Transferosome- Thin-layer evaporation technique Gel- Stirring method	Thyme (*Thymus vulgaris*) Oil and fluconazole (FO)	Transferosome- Size: 76.37 nm Zeta: −20.3 mV PDI: 0.233 EE: 52.38 ± 1.76% DL: % 9.09 ± 0.14, 1.43 ± 0.02 Gel- permeation flux: 4.101 μg/cm^2^/h Activity increases: 1.67-fold against *Candida albicans* compared to FO	Anti-fungal	[53]
Niosomal gel	Niosome- Cholesterol and surfactant, Gel- Carbopol	Niosome- Thin-film hydration technology Gel- Stirring method	Carvacrol oil	Niosome- Size: 180.23 nm Zeta: −31.70 mV PDI: 0.265 EE: 90.61% DR: 70.24 ± 1.21% Gel- Skin penetration: 25.0 µm	Anti-inflammation	[54]
Microgel	Chitosan and cinnamic acid	Ionic gelation	*Gaultheria procumbens* essential oil	EE: 65–70% DL: 30–35% DR: 26.66–88.33% 200 ppm inhibits: 17.85% aflatoxin B1 synthesis	Anti-microbial	[55]

**Table 3:** Different natural gelling agents and their applications.

Gelling Agent	Source	Binding Blocks	Pharmaceutical Application	Other Uses	Ref.
Pectin	Plant cell walls and fruits, e.g., apple, guava, and citrus fruit	(1 → 4)-α-D-galacturonic acid and natural sugar	Pectin-inorganic composite, pectin-organic polymer composite, and pectin-based hybrid materials (obtained by grafting reaction) for drug delivery	Food, agriculture, medicines, and biomedicine	[72]
Cellulose	-Wood pulp (cellulose nanocrystals and nanofibrils) *Acetobacter xylinum* (bacterial cellulose)	Homopolymer of D-glucose β-(1, 4)	Methylcellulose (MC), carboxymethylcellulose (CMC), ethyl cellulose (EC), hydroxyethyl cellulose (HEC), hydroxypropyl cellulose (HPC), and hydroxypropyl methylcellulose (HPMC) used as an excipient in different dosage forms	Biofilm, packaging, Implant, filtration, and composite	[73]
Agar	Gelatinous polysaccharides in the cell wall of many red algal species	Agarose (D-galactose and 3,6-anhydrous-L-galactose) and agaropectin (D- and L-galactose)	Hydrophobic modified agar for encapsulation and release of curcumin Agarose bioplastic for surgical and wound dressings Glutaric anhydride-modified agar for super-absorbent	Gelling, thickening, and stabilizing agent	[74,75,76,77]
Guar gum	Water-soluble polysaccharide derived from the seeds of *Cyamopsis tetragonolobus*, family Leguminosae	(1 → 4)-β-d-mannopyranosyl units with α-d-galactopyranosyl units attached by (1 → 6) linkages	Poly(acrylamide)-graft-guar gum for pH-sensitive microgel Hydrolyzed poly(acrylamide)-graft-GG for intestinal drug delivery	Film-forming and controlled drug-release abilities	[78]
Carrageenan (CG)	Red seaweeds of the Rhodophyceae class	Sulfated linear polysaccharide of D-galactose and 3, 6-anhydrous-d-galactose	Gelcarin^®^ GP-379 (τ-CG) and Viscarin^®^ GP-209 (λ-CG) for control drug delivery	Stabilizer in micro/nanoparticles systems and gelling agent	[79]
Carob gum or locust bean gum	Endosperm of the seeds of *Ceratonia siliqua* Linn.	Galactomannan (galactose and mannose)	Sodium carboxymethyl ether of locust bean gum for control drug release Acrylamide-grafted locust bean gum for controlled-release matrix tablet	Preparation of implants, films, beads, viscous liquid and gel formulations	[80,81,82]
Xanthan gum	Produced mainly by the bacterium *Xanthomonas campestris*	d-mannose (β-1,4), d-glucuronic acid (β-1,2) and d-mannose	Starch–xanthan gum for drug delivery Xanthan gum-based graft copolymers for water treatment and drug delivery	Emulsion stabilizer, texture modifiers,	[83,84]
Alginate	Brown marine algae, *Pseudomonas* sp. and *Azotobacter* sp.	Linear copolymeric blocks of l-guluronic acid and d-mannuronic acid residues.	Starch-modified alginate for drug delivery Sodium alginate for designing of nanocarrier	Stabilizer, thickening agent and emulsifying agent.	[85,86,87]
Gum Arabic	*Acacia nilotica*	Combination of saccharides and glycoproteins	pH-responsive matrix for colon-specific drug delivery Multi-aldehyde gum Arabic for anti-cancer drug delivery	Hard gummies, chocolates, and gums	[88,89,90]
Chitosan	Exoskeleton of crustacea, insect cuticles, algae and in the cell wall of fungi	N-acetyl D-glucosamine and 2-amino 2-deoxy-β-d-glucopyranose	Mannosylated chitosan for peptide delivery Trimethyl chitosan for fungal drug delivery	Water treatment, food processing, pharmaceutical/biomedicine, textiles and agriculture	[91,92,93,94]

Water vapor transmission rate (WVTR), moisture retention capability (MRC), minimum inhibitory volume (MIV), in vitro drug release (DR), ex vivo drug permeation (Dp), drug retention (Dr), drug loading (DL), entrapment efficiency (EE), zone of Inhibition (ZOI), not assessed (NA), amount of drug permeated (Q), drug flux (Jss), log permeability coefficient (Log Kp), simulated gastric fluid (SGF), simulated intestinal fluid (SIF), tensile strength (Ts).

**Table 4:** Potential patented herbal oil-based formulations of plant bioactives.

S. No	Patent/Application No.	Country	Title	Ref.
1	487445	India	Herbal gel formulation from the extract of curcumin and *annona muricata linn* and the manufacturing method	[168]
2	435835	India	An in situ gel formulation comprising carica papaya extracts for use in treatment of chronic periodontitis and method of synthesizing the same	
3	440711	India	A system and process for synthesizing oil-based liposomal gel for vitiligo and its composition	
4	438991	India	Liposomal gel formulation comprising 5-fluorouracil and clove oil for the treatment of skin cancer and method of preparation	
5	508000	India	Microemulgel composition comprising clove oil for the treatment of fungal infection and method of preparation	
6	492666	India	Formulation and evaluation of nano-emulgel containing *moringa oleifera* seed oil for wound healing	
7	445175	India	Pharmaceutical composition with synergistic combination of thymus serphyllum essential oil and antibiotic	
8	471183	India	Formulation comprising ozonized oil in the treatment of a tumor	
9	505804	India	Tretradydrocurcumin niosomal in situ gel for ocular drug delivery	
10	424456	India	Pharmaceutical composition of thymoquinone and essential oil particulate emulsified systems	
11	201821031701	India	Herbal in situ gel implant comprising thyme oil	[169]
12	202311051599	India	A topical lumpy skin disease (lsd) wound-healing herbal gel and preparation method	
13	202141025017	India	Herbal gel for treatment of acne	
14	202221059726	India	Herbal gel formulation for acne treatment and preparation method	
15	202111003298	India	Herbal nanoemulsion gel and a process for the preparation	
16	ES2885052T3	Spain	Multifunctional formulation composed of natural ingredients and its preparation/manufacturing method	[170]
17	CN103262839B	China	Mosquito-repelling gel composition with natural plant essential oil	[171]
18	US20180311184A1	US	Topical analgesic pain relief formulations, manufacture and use	[172]
19	US9717240B2	US	Applications of microencapsulated essential oils	[173]
20	US10542760B2	US	Skin and surface disinfectant compositions containing botanicals	[174]

## Data Availability

Not applicable.
